# Identification and Characterization of the Core Region of *ZmDi19-5* Promoter Activity and Its Upstream Regulatory Proteins

**DOI:** 10.3390/ijms23137390

**Published:** 2022-07-02

**Authors:** Yang Zhao, Lijuan Xu, Yuanxiang Huang, Hongying Wu, Xingen Zhang, Xiaolin Hu, Qing Ma

**Affiliations:** National Engineering Laboratory of Crop Stress Resistance Breeding, School of Life Sciences, Anhui Agricultural University, Hefei 230036, China; zhaoyang2013@ahau.edu.cn (Y.Z.); m18355093629@163.com (L.X.); 13696549319@163.com (Y.H.); why38762022@163.com (H.W.); 13145518767@163.com (X.Z.); huxiaolin6899@163.com (X.H.)

**Keywords:** maize, ZmDi19-5, drought stress, promoter, regulatory proteins

## Abstract

Drought-induced 19 (Di19) family genes play important roles in plant growth, development, and environmental stress responses. However, little is known about this family in maize. The upstream regulatory network of Di19 genes remains poorly understood in plant stress response, especially. In this study, seven ZmDi19 genes were identified, and sequence alignment, gene structure, and phylogenetic analysis was conducted. According to the phylogenetic analysis, the *ZmDi19-5* promoter was cloned and multiple putative stress-responsive *cis*-acting elements (CAEs) were found in the promoter region. The transient transformation assay indicated that firefly luciferase (LUC)-expressed activity driven by the *ZmDi19-5* promoter can be significantly induced by drought stress. A 450 bp core region of *ZmDi19-5* promoter was identified, and 28 upstream regulatory proteins were screened using yeast one-hybird (Y1H) system. According to the functional annotation, some genes were related to photosynthesis, light response, and water transport, which may suggest the important roles of these genes in drought response. Particularly, five members that may be involved in drought response exhibited strong binding activity to the core region of the *ZmDi19-5* promoter. This study laid an important foundation for further revealing the molecular mechanisms and regulatory network of Di19 genes in drought stress response.

## 1. Introduction

Drought, high salinity, and extreme temperature are the most common abiotic stresses that affect plant growth and crop productivity worldwide [1,2]. During the long period of evolution, multiple response mechanisms including molecular, cellular and physiological changes have been established to adapt these adverse effects of environmental stresses [3,4]. Among them, a major strategy is that a variety of stress-related genes are induced and the levels of stress resistance-related functional proteins are accumulated under stressed conditions, which can directly or indirectly participate in plant stress signal transduction and stress response [2,5,6]. Transcription factors (TFs) are extensively involved in the regulation networks of plant growth and development, which can activate or repress the expression of target genes through binding to the promoter regions [7,8]. TFs have also been known to play important roles in plant response to various abiotic stresses, which can be divided into different gene families, such as bZIP, WRKY, NAC, AP2/EREBP, and MYB, according to their DNA-binding domain [9,10]. Regulating the expression of TFs to improve plant tolerance to various abiotic stresses has been reported in many studies [11,12,13,14,15].

Drought-induced (Di19) proteins play important roles in plant growth and stress response, which belong to Cys2/His2-type (C2H2) zinc-finger protein family [16,17,18]. The typical characters of C2H2 zinc-finger proteins is to contain one or more tandem of Cys2/His2 zinc-finger domains, which are encoded by one of the largest transcription factor families in eukaryotes [19,20,21]. Studies have indicated that Di19 proteins contain two unusual Cys2/His2 zinc-finger motifs, which are highly conserved in evolution [22]. Usually, a di19_C domain is also found in the C terminal of Di19 protein. Unlike with the classical zinc-finger protein families, the Di19 proteins are encoded by a small gene family. For example, a total of seven Di19 proteins were identified in the Arabidopsis genome [16], and the same number of proteins was found in rice [18] and soybean [23].

Expression pattern analysis indicated that most Di19 genes can be regulated by various abiotic stresses [16,18,23]. It was reported that *AtDi19-1* and *AtDi19-3* were significantly induced under drought stress, whereas expression of *AtDi19-2* and *AtDi19-4* were significant induced by high salinity [16]. Further functional analysis was also performed for these genes in plants. For example, overexpression of *AtDi19-3* reduced tolerance of the transgenic plants to drought and salt stresses. In rice, expression levels of *OsDi19-3* and *OsDi19-4* were rapidly up-regulated by osmotic stress and salt stress, whereas overexpression of *OsDi19-4* leads to drought tolerance phenotype of transgenic rice and enhanced reactive oxygen species (ROS)-scavenging activity [18]. In soybean, *GmDi19-5* and *GmDi19-6* showed increased expression under drought treatment; overexpression of *GmDi19-5* increased sensitivity of transgenic plants to various stresses [23]. Studies also indicated that the expression of abscisic acid (ABA) and salt overly sensitive (SOS) signal pathway related genes was altered in the Di19 overexpressing transgenic lines, suggesting that Di19 genes were involved in ABA and SOS signaling pathway [22,23,24]. Di19 proteins can interact with calcium-dependent protein kinases (CPKs) and are phosphorylated by CPKs. For example, AtCPK11 can enhance the transactivation activity of AtDi19, which can directly up-regulate the expression of PR1 (pathogenesis-related), PR2, and PR5 in response to drought stress [17]. Overexpression of cotton *GhDi19-1* and *GhDi19-2* in Arabidopsis enhanced plant sensitivity to high salinity and exogenous ABA [25]. Further analysis indicated that serine site is crucial for the functionally activating of GhDi19-1 and GhDi19-2 in salt response and ABA signaling, which may function as downstream targets of CDPKs in ABA signal pathways [26].

As mentioned above, increasing evidence has indicated that Di19 proteins play important roles in response to abiotic stresses. A promoter is an important regulatory sequence that drives the expression of downstream genes, and the contained *cis*-acting elements have a greater impact on the transcriptional regulation of downstream genes involved in various biological processes [27,28,29]. Analyzing the structural characteristics of a gene promoter, thus identifying the upstream regulatory proteins is of great significance to explore the molecular mechanisms and regulatory network of Di19 genes in abiotic stress responses. In this study, we performed a systematical analysis of Di19 gene family in maize, including sequence structure, conserved motif, chromosomal location, phylogenetic relationship, etc. According to the results of bioinformatic analysis, the *ZmDi19-5* promoter was cloned and its activity analysis under drought stress was performed. Importantly, upstream regulatory proteins of *ZmDi19-5* were screened using the yeast one-hybrid (Y1H) system with the identified core region of the promoter activity.

## 2. Results

### 2.1. Identification of ZmDi19 Genes

**Figure 1:**
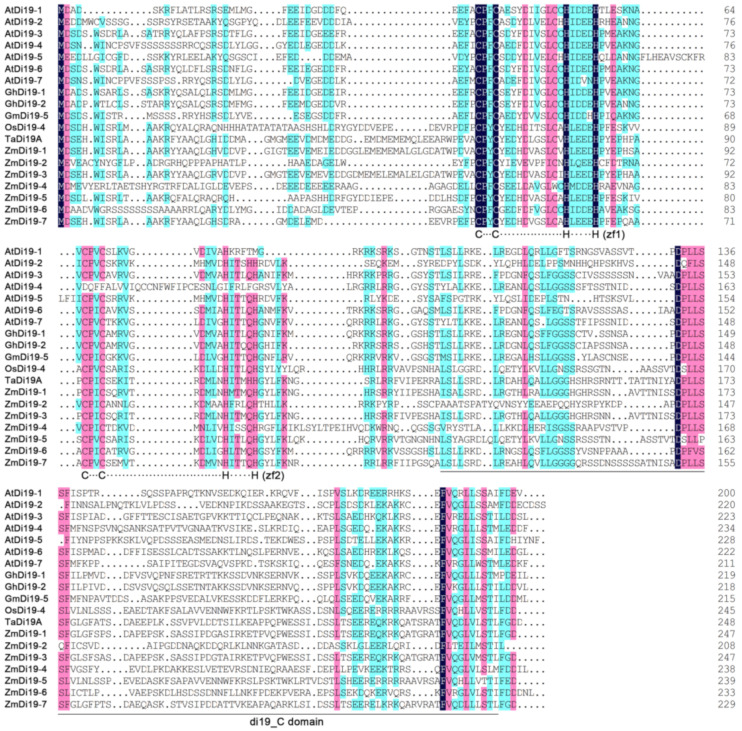
Sequence alignments of maize, Arabidopsis, and other Di19 members with known functions. Sequences were aligned by DNAMAN software, and identical or similar residues were highlighted in different colors.

The HMM profiles of the zf-Di19 and Di19_C domains were used to search the Di19 family genes in maize using BLASTP program, and candidate sequences containing both of the two domains were considered to be a Di19 family gene. Finally, a total of seven non-redundant Di19 proteins were identified, and were randomly named as ZmDi19-1–ZmDi19-7. In addition, a total of 6 sorghum Di19 members (SbDi19-1–SbDi19-6) were downloaded from the phytozome database and subsequently used for phylogenetic analysis. The total number of ZmDi19 genes was the same with that in Arabidopsis and rice [16,18]. Sequence alignments indicated that all of the ZmDi19 sequences contained the two atypical Cys2/His2 zinc-finger motifs in the zf-Di19 domain. These results suggested that ZmDi19 genes are highly conserved in evolution, consistent with the observations of Di19 sequence characteristics in other species (Figure 1). The amino acid lengths of ZmDi19 proteins were ranged from 208–247 amino acids. The molecular weights and isoelectric points of ZmDi19 proteins ranged from 23.49 to 27.33 kDa and 4.55 to 6.67, respectively. The detailed information of ZmDi19 proteins was shown in Table S1.

### 2.2. Phylogenetic Relationships, Gene Structure, Chromosomal Location, and Gene Duplication

**Figure 2:**
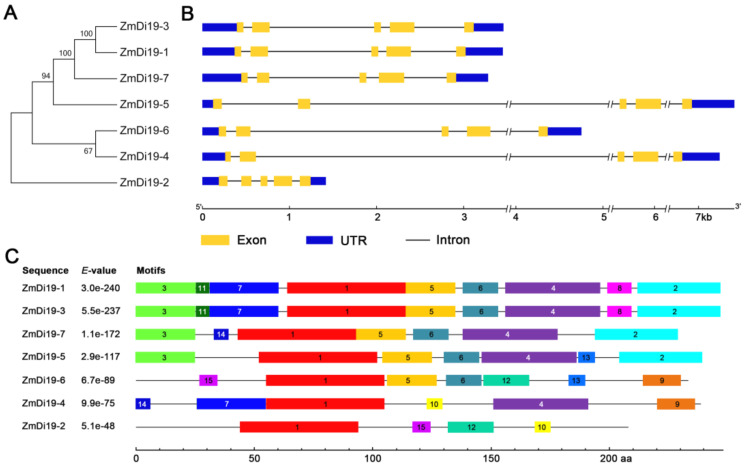
Phylogenetic tree, gene structure, and motif composition of ZmDi19 proteins. (**A**) Unrooted neighbor-joining phylogenetic tree of the ZmDi19 proteins. (**B**) Exon–intron structures of the ZmDi19 genes. Exons are shown as yellow boxes, introns are shown as black lines, and un-translated regions (UTRs) are shown as blue boxes. (**C**) Distribution of conserved motifs in the ZmDi19 proteins.

To explore the phylogenetic relationships of ZmDi19 genes, an unrooted phylogenetic tree of the seven sequences was constructed on the basis of multiple sequence alignments using the full-length amino acid sequences via the Neighbor-Joining (NJ) method. *ZmDi19-3*/*ZmDi19-1* and *ZmDi19-6*/*ZmDi19-4* formed two gene pairs, implying their close evolutionary relationships (Figure 2A). The exon-intron analysis indicated that ZmDi19 genes shared similar gene structure, and all of them have five exons. We noted that ZmDi19 genes were conserved in the distribution and length of exons, but had a large variation in the intron length (Figure 2B). To further reveal the sequence characteristics of ZmDi19 members, a total of 15 conserved motifs were identified among the ZmDi19 proteins by the Multiple Expectation Maximization for Motif Elicitation (MEME) online tool. Motif 1 encoding the zf-Di19 domain was highly conserved, which was identified in each of the ZmDi19 proteins. Motif 2 encoding the Di19_C domain was identified in ZmDi19-1, ZmDi19-3, ZmDi19-7, and ZmDi19-5 (Figure 2C). In addition, motifs 4, 5, and 6 were identified in most of the ZmDi19 proteins. We found the motif distribution of gene pair *ZmDi19-3*/*ZmDi19-1* was closely related with the phylogenetic relationship and exon/intron structure, which might suggest their putative redundant functions. Detailed information about the conserved amino acid sequences of the 15 motifs was presented in Table S2.

**Figure 3:**
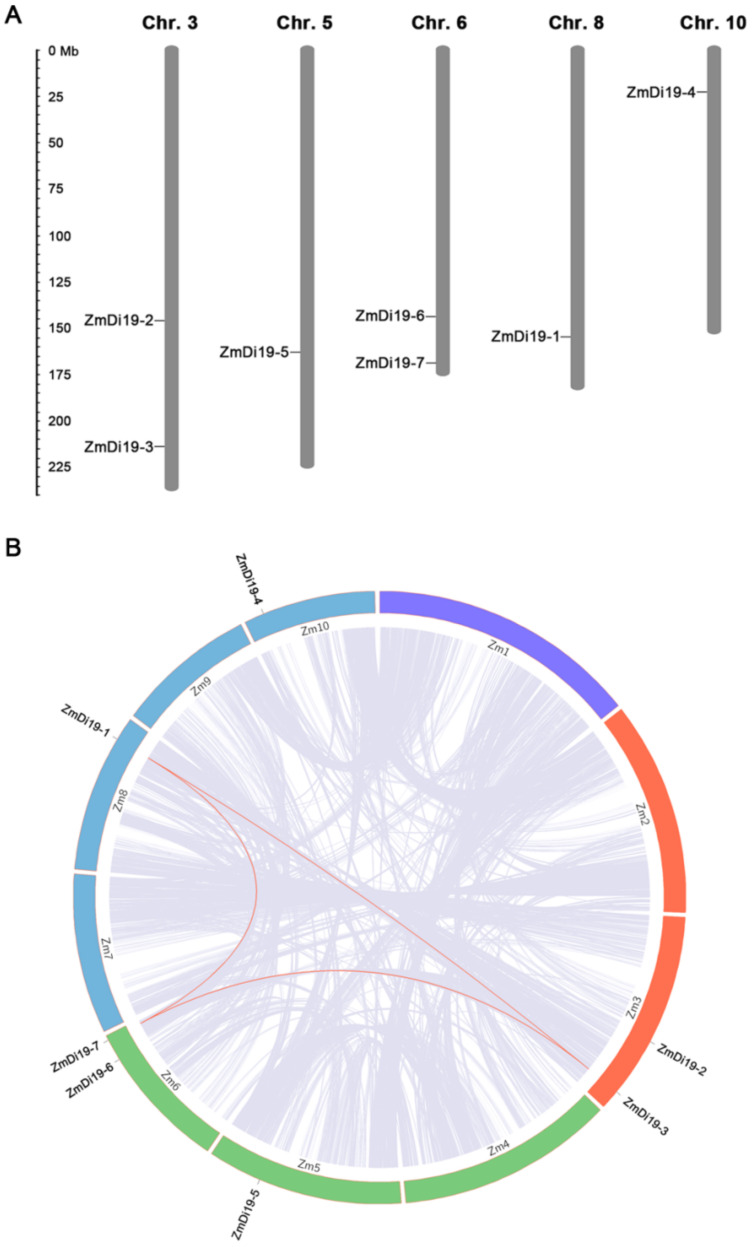
Chromosomal location and syntenic analysis of ZmDi19 genes. (**A**) Seven ZmDi19 genes located on the five of the 10 chromosomes. (**B**) Syntenic analysis among ZmDi19 genes.

Chromosomal location analysis indicated that ZmDi19 genes were located on five of the 10 chromosomes. *ZmDi19-2* and *ZmDi19-3* were located on chromosome 3, *ZmDi19-6* and *ZmDi19-7* were located on chromosome 6. *ZmDi19-5*, *ZmDi19-1*, and *ZmDi19-4* were located on chromosomes 5, 8 and 10, respectively (Figure 3A). In addition, three ZmDi19 genes (*ZmDi19-1*, *ZmDi19-3* and *ZmDi19-7*) were located on the segmental duplication regions based on the syntenic regions and phylogenetic analysis, and no tandem duplication was identified (Figure 3B).

**Figure 4:**
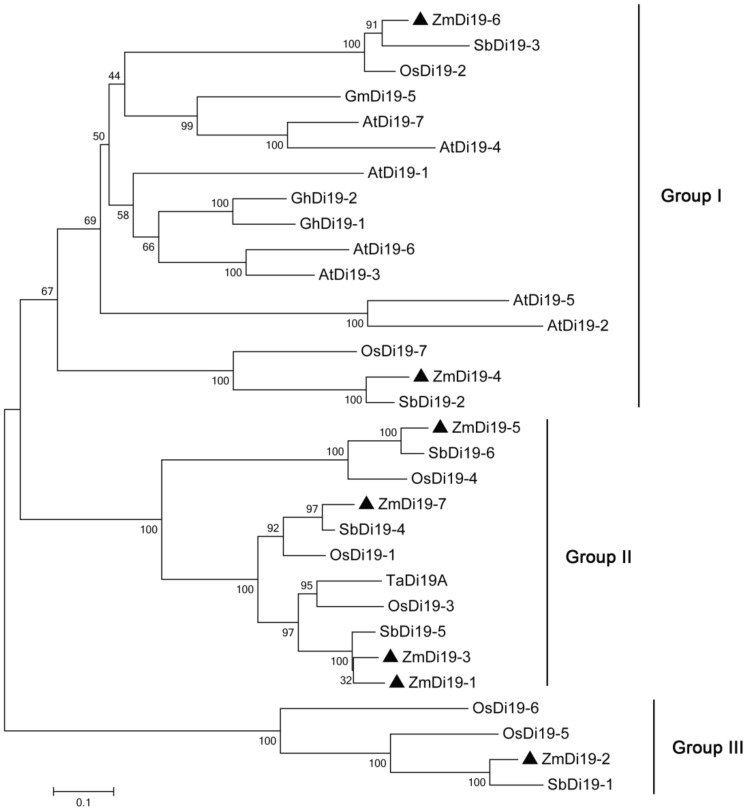
Phylogenetic tree of Di19 proteins from maize, rice, Arabidopsis, and other species.

To further examine the phylogenetic relationships of Di19 gene family among different species, a total of 31 Di19 protein sequences from maize, rice, sorghum, Arabidopsis, and other species with known functions, were used to construct a combined phylogenetic tree. As shown in Figure 4, the 31 Di19 proteins were divided into three groups (I–III). Group I and II constituted the largest clades, which contained 16 and 11 Di19 proteins, respectively, and group III contained the least number of proteins (4). We noted that the Di19 proteins from monocots and dicots were clustered into different groups. All of the Di19 proteins from dicots were clustered in Group I, which also contained 6 proteins from monocots (2 each from maize, rice and sorghum). Groups II and III contained only Di19 proteins from monocots, suggesting the evolutionary divergence of Di19 gene family in different species. Some ZmDi19 proteins showed close relationships with the known functional genes; for example, *ZmDi19-5* and *OsDi19-4* were clustered in the same clade, which indicated the important roles of these genes in stress responses.

### 2.3. Expression Pattern Analysis of ZmDi19 Genes at Different Developmental Stages

**Figure 5:**
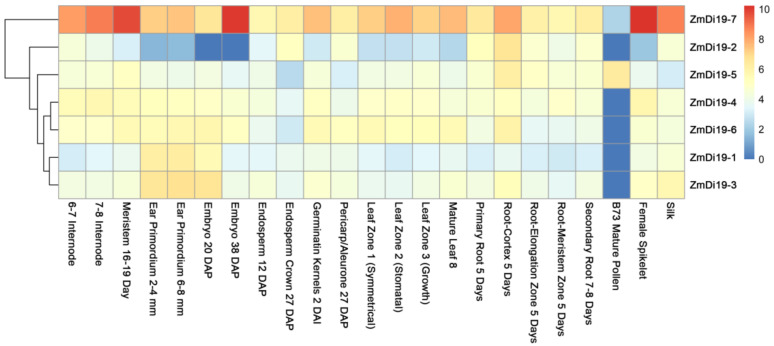
Expression profiles of ZmDi19 genes at different developmental stages. The color scale at the right of the figure indicates the FPKM normalized log2 values, and the heatmap was drawn using R package pheatmap.

To investigate the tissue expression profiles of ZmDi19 genes, public RNA-seq data across different developmental stages was downloaded from the MaizeGDB database provided by [30], and heatmap analysis was performed based on the log_2_ (FPKM + 1) values. The RNA-seq data cover 23 developmental stages throughout the entire life cycle of maize. Results indicated that the ZmDi19 genes have differentially expression patterns across different developmental stages, and no members showed tissue-specific expression among the detected tissues (Figure 5). We noted that *ZmDi19-7* was highly expressed in most of the developmental stages compared with other ZmDi19 genes, and all of the ZmDi19 genes have relatively low expression levels in mature pollen with the exception of *ZmDi19-5*. For the three genes involved in segmental duplication, *ZmDi19-1* and *ZmDi19-3* exhibited similar expression patterns, which might suggest their redundant functions during the plant growth and development.

### 2.4. Sequence Analysis and Expressed Activity of ZmDi19-5 Promoter under Drought Stress

**Figure 6:**
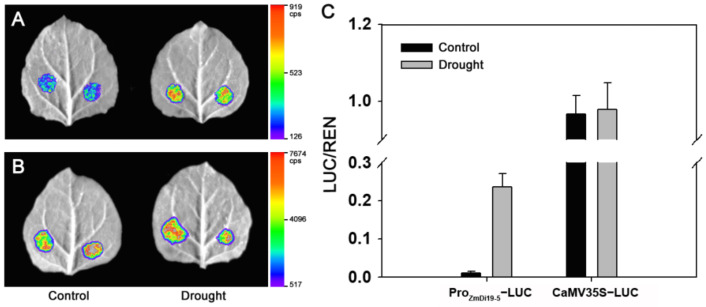
LUC expressed activity of *ZmDi19-5* promoter under control and drought conditions. (**A**) LUC expressed activity driven by *ZmDi19-5* promoter; (**B**) LUC expressed activity driven by 35S promoter; (**C**) Quantitative analysis of LUC activity under control and drought conditions.

Previous studies have demonstrated that *OsDi19-4* plays an important role in rice drought response, and GUS activity driven by the *OsDi19-4* promoter can be induced under stress treatments [18]. Since *ZmDi19-5* exhibited a close phylogenetic relationship with *OsDi19-4*, the 2000 bp promoter region of *ZmDi19-5* was cloned from inbred line B73 to understand the regulatory mechanism of ZmDi19 genes involved in drought response. The cloned sequence of the *ZmDi19-5* promoter showed the same nucleotide sequences with the sequence in the reference genome of maize (AGPv4). The promoter sequence was analyzed by PlantCARE database, and five types of stress-related *cis*-acting elements (CAEs) were investigated, including ABA-responsive elements (ABREs), dehydration-responsive elements (DRE), low-temperature-responsive element (LTRE), MYB elements, and MYB binding site involved in drought-inducibility (MBSs) involved in drought response. Multiple stress-related CAEs were found in the promoter regions, including 3 ABREs, 2 DRE core elements, 1 DRE1, 1 LTR, 4 MYBs, and 2 MBSs (Figure S1). To confirm these CAEs, the results were compared with the binding sites predicted by the PlantRegMap database. We found that most of the CAEs identified by PlantCARE were contained in the results predicted by the PlantRegMap database although some differences were also found between the two databases (Table S3). Subsequently, *Agrobacterium*-mediated transient transformation was adopted to analyze the firefly luciferase (LUC) expression in tobacco to investigate the *ZmDi19-5* promoter activity. Results indicated that the LUC-expressed activity can be normally detected under the controlling of the *ZmDi19-5* promoter, but significantly lower than the activity controlled by the cauliflower mosaic virus (CaMV) 35S promoter. To investigate whether the *ZmDi19-5* promoter activity can be regulated by drought stress, the tobacco plants were irrigated with 20% PEG for 1d before *Agrobacterium* transformation. As shown in Figure 6A, the LUC activity was significantly higher under drought treatment than that of the normal conditions. However, no significant difference was observed for the LUC activity driven by the 35S promoter between drought and normal conditions (Figure 6B,C). These results indicated the activity of the *ZmDi19-5* promoter can be induced by drought stress.

### 2.5. Identification of Core Region of ZmDi19-5 Promoter

**Figure 7:**
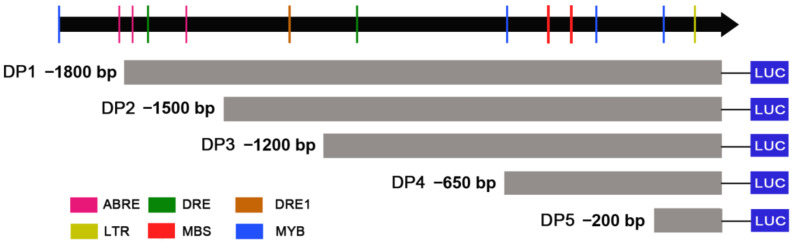
Schematic diagram of the different truncated fragments of *ZmDi19-5* promoter.

**Figure 8:**
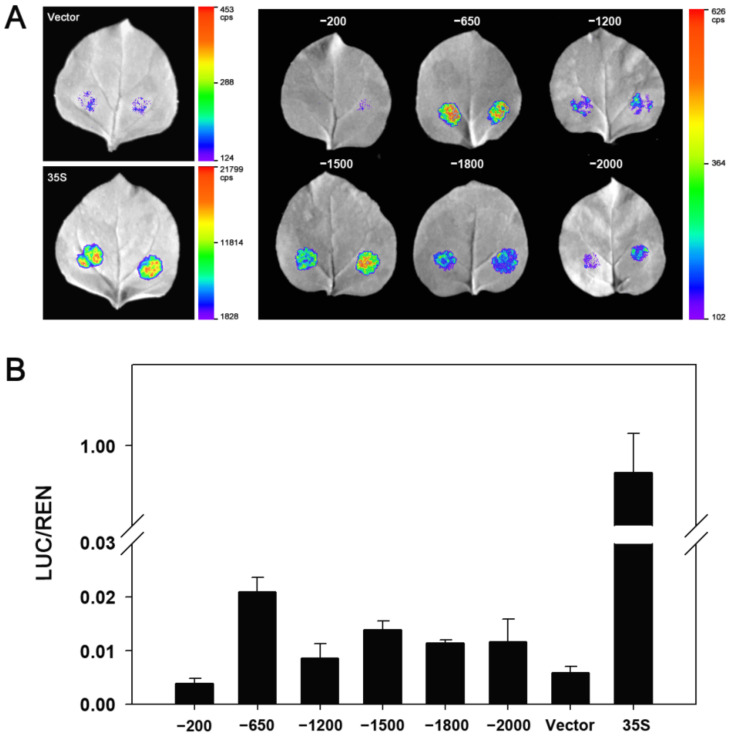
Identification of the core region of *ZmDi19-5* promoter. (**A**) LUC-expressed activity driven by different truncated *ZmDi19-5* promoter, and 35S:LUC construct (35S) and pGreenII 0800-LUC empty vector (Vector) were used as the controls, respectively; (**B**) Quantitative analysis of LUC-expressed activity.

According to the CAEs distributions, five truncated fragments from 5′terminal were obtained using PCR amplification to identify the core region of the *ZmDi19-5* promoter activity. The length of the five truncated fragments were: −1800 bp (DP1), −1500 bp (DP2), −1200 bp (DP3), −650 bp (DP4), and −200 bp (DP5), respectively (Figure 7). Each of the five truncated fragments was cloned into the pGreenII 0800-LUC vector to drive LUC expression. Then, the constructed vector harboring the truncated fragment and the full length (2000 bp) of the *ZmDi19-5* promoter were transformed into tobacco with the same method of *Agrobacterium*-medicated transient transformation, respectively. The pGreenII 0800-LUC empty vector and constructed 35S:LUC vector were also transformed as the experimental controls, respectively. With the exceptions of DP5 construct and pGreenII 0800-LUC empty vector, significant LUC activity can be detected in each of the vector transformations (Figure 8A). According to the quantitative results, the detected LUC activity in the transformation of the DP4 construct was the strongest, and the LUC activity was dramatically decreased in the transformation of DP5 construct, suggesting that the 450 bp (−200 to −650) fragment was the core region required for the expressed activity of the *ZmDi19-5* promoter (Figure 8B). Sequence analysis revealed that the 450 bp fragment contained 2 MBS and 2 MYB CAEs, which mays be related to drought response.

### 2.6. Identification of Upstream Regulatory Proteins of ZmDi19-5

**Figure 9:**
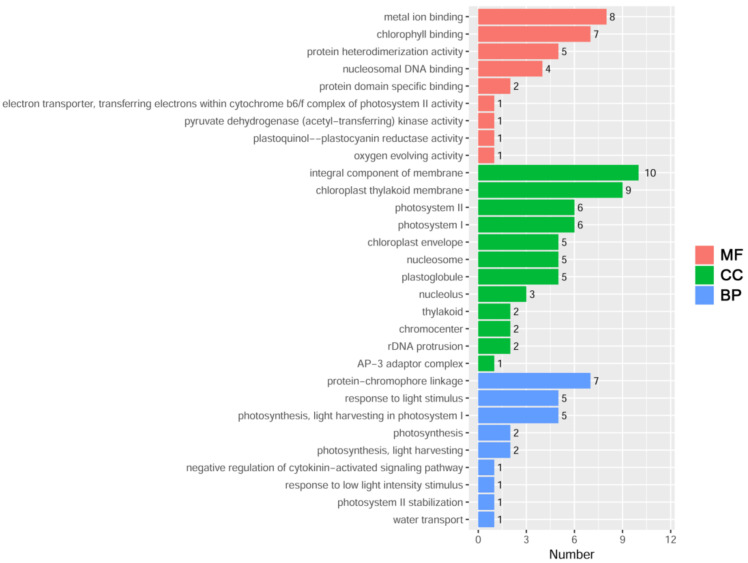
GO enrichment analysis of the 28 screened upstream regulatory factors of *ZmDi19-5*.The 30 most significant GO terms are shown in the figure. MF, molecular function; CC, cellular component; BP, biological process.

To identify the upstream regulatory proteins of *ZmDi19-5*, the 450 bp core region of the *ZmDi19-5* promoter was cloned into a pAbAi vector to construct a pAbAi-Pro_ZmDi19-5_-450 vector, and transformed into yeast Y1HGold strain. Then, the 450 bp core region was used to screen the maize leaf cDNA library using the Y1H system. After the screening, a total of 28 sequences were obtained through yeast plasmid isolation and nucleotide sequencing (Table S4). To further explore the biological functions of the screened proteins, Gene Ontology (GO) enrichment analysis was performed for these protein sequences, and the results indicated that the 28 sequences were significantly assigned to 45 GO categories (*p*-value < 0.05), including 18 biological processes (BP), 14 cellular components (CC), and 13 molecular function (MF) terms (Figure 9). Most genes were assigned into GO terms of the integral component of the membrane (GO:0016021), chloroplast thylakoid membrane (GO:0009535), metal ion binding (GO:0046872), chlorophyll binding (GO:0016168), protein-chromophore linkage (GO:0018298), photosystem I (GO:0009522), photosystem II (GO:0009523), photosynthesis, light harvesting in photosystem I (GO:0009768), plastoglobule (GO:0010287), nucleosome (GO:0000786), response to light stimulus (GO:0009416), protein heterodimerization activity (GO:0046982), and chloroplast envelope (GO:0009941). We found that photosynthesis and light response-related terms were the main types among the 18 BP terms. In addition, one gene was assigned in the water transport (GO:0006833) and negative regulation of cytokinin-activated signaling pathway (GO:0080037) terms, respectively.

**Figure 10:**
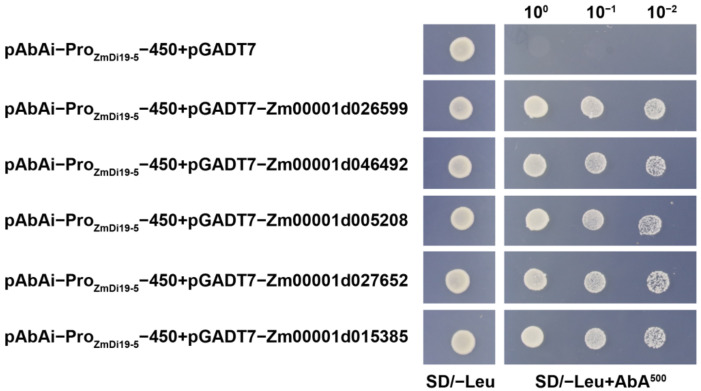
Binding activity of five important proteins to the core region of *ZmDi19-5* promoter by Y1H.

According to the functional annotations of screened regulatory factors as well as Arabidopsis and rice homologs, five members, including Zm00001d026599 (light harvesting chlorophyll a/b binding protein), Zm00001d046492 (heme oxygenase), Zm00001d005208 (NAC transcription factor), Zm00001d027652 (tonoplast intrinsic protein), and Zm00001d015385 (chlorophyll a/b binding protein), were selected for further verification using the Y1H system. The five members or their homologous were mainly involved in the biological processes of response to abiotic stresses, photosynthesis, and hormone response. As shown in Figure 10, all of the positive clones harboring the identified regulatory factors and the negative control (pAbAi-Pro_ZmDi19-5_-450 + pGADT7) can grow on the SD/-Leu plates after being cultured at 30 °C for 3 d. When the clones were inoculated on the SD/-Leu plates containing 500 ng/mL Aureobasidin A (AbA), we found that the detected clones harboring the identified regulatory factors could grow normally, but the negative control could not grow, further confirming the binding ability of the screened proteins to the core region of the *ZmDi19-5* promoter.

## 3. Discussion

Transcription factors constitute multiple gene families in the plant genomes, which play indispensable roles in plant growth, development, and abiotic stress response [31]. Di19 proteins are of low molecular weight, encoded by small multi-genes in plants. Studies indicated that Di19 transcription factors were involved in abiotic stress responses. Although Di19 gene family have been extensively analyzed in Arabidopsis, rice, soybean, and other species [16,18,23,24], a systematic characterization of Di19 family genes in maize has not been reported as yet. Although the biological functions of Di19 family members have been reported in stress response in some previous studies, little is known about the regulatory network of this type of gene. In the present study, a total of seven ZmDi19 proteins were identified in the current maize genome. Interestingly, although the maize genome size is significantly larger than that of Arabidopsis and rice, the same number of Di19 genes (7) was found in these species, and the number of ZmDi19 family was similar with that of in sorghum (6). It is well known that gene duplications, including tandem and segmental duplications, are one of the important driving forces in the genome expansion during the process of plant evolution [32]. Previous studies indicated that tandem duplications are common in the large and rapidly evolving gene families, whereas segmental duplication occurred more often in slowly evolving gene families [33]. Based on the syntenic analysis and phylogenetic relationships, three ZmDi19 genes (*ZmDi19-1*, *ZmDi19-3*, and *ZmDi19-7*) were involved in the segmental duplications, whereas no tandem duplication was found in ZmDi19 family, suggesting that the Di19 gene family is a highly conserved and slowly evolved gene family in plants, which might be responsible for similar family members in different plant species.

As mentioned above, Di19 proteins belong to C2H2 zinc-finger protein transcription factors [16,17,18]. The Pfam analysis indicated that each of the ZmDi19 proteins contain both of the zf-Di19 and Di19_C domains. Two atypical zinc-finger motifs, Cys-X_2_-Cys-X_11_-His-X_4_-His and Cys-X_2_-Cys-X_10_-His-X_4_-His, are especially highly conserved in the zf-Di19 domain. ZmDi19 proteins are similar in the distribution of protein motifs and gene structure. For example, each of the ZmDi19 genes contained five exons. These observations further confirmed the conserved evolution of Di19 gene family in plants. Based on phylogenetic analysis, the 31 Di19 proteins from maize, rice, sorghum, Arabidopsis, and other reported members were divided into three groups with high bootstrap values. We found the different evolutionary characteristics of Di19 gene family between dicot and monocot species. A total of six proteins from monocots were clustered in group I, and no monocot Di19 proteins were contained in groups II and III. It should be noted that some ZmDi19 members exhibited closely relationships with their orthologs in sorghum and rice, indicating that these genes might have been generated from a common ancestor, such as *ZmDi19-4*, *OsDi19-7*, and *SbDi19-2* in group I, and *ZmDi19-5*, *OsDi19-4*, and *SbDi19-6* in group II. To date, only a few Di19 genes have been reported in monocots, such as *OsDi19-4*, *ZmDi19-1*, and *TaDi19A* [18,24,34]. We found that *ZmDi19-5* has a close phylogenetic relationship with *OsDi19-4*, and *ZmDi19-7* shares a close phylogenetic relationship with *TaDi19A*.

In previous studies, crucial biological functions of Di19 family genes have been presented in the regulating of stress responses. For example, *AtDi19-1* overexpressing plants have shown improved tolerance to drought stress compared with WT plants. On the contrary, overexpression of *AtDi19-3* increased the sensitivity of transgenic Arabidopsis plants to drought, salt stresses, and exogenous ABA [17,22]. Transgenic Arabidopsis plants overexpression *GmDi19-5* exhibited increased sensitivity to salt, drought, and oxidative and ABA stresses [23]. According to the phylogenetic analysis, *ZmDi19-5* exhibited a close phylogenetic relationship with *OsDi19-4* in group II. Previous studies have shown that GUS activity driven by the *OsDi19-4* promoter can be induced by salt stress and dehydration in tobacco epidermal cells, and overexpression of *OsDi19-4* increased the tolerance to drought stress in transgenic plants [18]. To investigate the promoter activity of ZmDi19 genes in response to drought stress, the promoter activity of *ZmDi19-5* was investigated through *Agrobacterium*-medicated transient transformation by controlling LUC expression. We found the promoter activity of *ZmDi19-5* can be significantly induced under drought treatment, indicating the crucial roles of *ZmDi19-5* in drought response. Further analysis was performed to identify the core region of the *ZmDi19-5* promoter activity through 5′terminal truncated experiments. We found that the DP4 construct (−650 bp to −1) exhibited the highest expressed activity among the six transformations, but the DP5 (−200 to −1) barely detected LUC activity. Therefore, the 450 bp (−650 bp to −200 bp) were considered to be the core region of *ZmDi19-5* promoter activity. Previous studies indicated that the MBS element is important for drought induced expression of the *ZmSO* promoter [35], and we found that the 450 bp core region contained two putative MYB and MBS elements, which might suggest the important regulatory roles of *ZmDi19-5* promoter expression in drought response.

Further investigating the upstream regulatory proteins thus enriches the regulatory network of the Di19 family genes involved in abiotic stress responses. A total of 28 upstream regulatory factors were obtained through Y1H screening using the bait of the 450 bp core region of the *ZmDi19-5* promoter. GO annotation indicated that some genes were enriched in photosynthesis, light response, and water transport-related biological processes. Previous studies indicated that photosynthesis is one of the main processes that is affected by drought stress, and numerous genes can be regulated during this process [36,37], suggesting that the related regulatory proteins play important roles in the regulation of plant photosynthesis under drought stress. Five regulatory proteins were especially chosen for further verification by the Y1H method, including Zm00001d026599, Zm00001d046492, Zm00001d005208, Zm00001d027652, and Zm00001d015385, demonstrated strong binding activity to the 450 bp core region. Although no direct function of these genes related to drought stress has been reported in maize, their expression or orthologs are closely related to various abiotic responses. Zm00001d026599 encodes a chlorophyll a/b binding protein, and a recent study has shown that expression of lhcb6 could be significantly regulated after the application of poly-γ-glutamic acid, which can significantly enhance drought resistance in maize [38]. The Arabidopsis homologs of Zm00001d046492 have been shown to play important roles in salinity responses [39]. Zm00001d005208 encodes a NAC (NAM, ATAF and CUC) transcription factor, and the rice homolog *SNAC1* has been demonstrated to improve the tolerance to drought and salt stresses in transgenic plants [40]. *OsAQP*, a rice homolog of Zm00001d027652, encodes a tonoplast intrinsic protein (TIP) of the aquaporin family, and expression of *OsAQP* can be regulated by drought, salinity, and cold as well as phytohormones [41]. Zm00001d015385 encodes a chlorophyll a/b binding protein, and the Arabidopsis homolog *AtLHCA1* has been shown to be involved in cold and heat stress responses [42]. Taken together, these results indicated the five members may be the important upstream regulators of *ZmDi19-5* in the regulation of drought stress response. However, we should also note that the functions of many genes are unknown in maize; therefore, the upstream regulatory proteins involved in the regulation of *ZmDi19-5* may not be limited to these.

## 4. Materials and Methods

### 4.1. Identification and Sequence Analysis of Di19 Genes in Maize

The maize genome and protein database (RefGen_v4) was downloaded from the Ensemble database (ftp://ftp.ensemblgenomes.org/pub/plants/release-41/fasta/zea_mays/, accessed on 29 March 2019). The Hidden Markov Model (HMM) profiles of the zf-Di19 (PF05605) and Di19_C (PF14571) were retrieved from the Pfam database (http://pfam.xfam.org/, accessed on 8 April 2019) [43], and were adopted as queries against maize genome protein database to identify maize Di19 proteins. The candidate maize Di19 protein sequences were confirmed for the two domains using the Pfam database, and only proteins containing both of the zf-Di19 and Di19_C domains were considered as Di19 family proteins. Finally, all of the candidate sequences were aligned using ClustalW, and redundant members were removed [44]. The Arabidopsis Di19 family proteins [16] were retrieved from the TAIR (http://www.arabidopsis.org/, accessed on 9 April 2019), rice Di19 protein sequences [18] were downloaded from the RGAP (http://rice.plantbiology.msu.edu/, accessed on 9 April 2019), and sorghum Di19 protein sequences were downloaded from Phytozome v12 (https://phytozome.jgi.doe.gov/, accessed on 18 April 2019). Bioinformatic analysis, including sequence characteristic, gene structure, conserved motif, chromosomal location, and phylogenetic analysis, were performed according to our previous study [45]. To conduct syntenic analysis, we used MCScanX package to identify synteny blocks within the maize genome to further detect the duplicated genes according to previous studies [46,47]. The complete Di19 protein sequences of maize, rice, Arabidopsis, and other known proteins were aligned using DNAMAN software.

### 4.2. Phylogenetic Analysis

To construct the unrooted phylogenetic tree, the full amino acid sequences of the maize Di19 proteins were aligned using the ClustalW program in the MEGA 7.0 software with default parameters [48]. The phylogenetic tree was constructed with the NJ method, and bootstrap analysis was conducted with 1000 replicates with the pairwise deletion option. The phylogenetic tree of the Di19 proteins from maize, rice, sorghum, Arabidopsis and other species was constructed with the same method to explore the phylogenetic relationships within different species.

### 4.3. Expression Pattern Analysis Using Transcriptome Data

To determine the expression patterns of maize Di19 genes, the RNA-seq data of maize 23 tissues of different vegetative and reproductive stages was downloaded from MaizeGDB provided by [30]. The FPKM value was used to represent the expression levels of each gene, and the log_2_ (FPKM + 1) value was calculated to generate the heat map using R package pheatmap as described in our previous study [45].

### 4.4. Cloning and Sequence Analysis of ZmDi19-5 Promoter

The 2000 bp promoter sequence upstream from the transcription start site of *ZmDi19-5* was obtained from the Phytozome database according to the maize genome annotation. To isolate the promoter, genomic DNA from leaves of maize inbred line B73 was extracted, and used as a template for PCR amplification with specific primers (ZmDi19-5pro-F and ZmDi19-5pro-R, Table S5). The purified PCR product was first cloned to pEASY^®^-Blunt Simple Cloning Vector (TransGen Biotech Co., Ltd., Beijing, China) for sequencing verification, and aligned with the sequences in the maize reference genome (RefGen_v4) with MEGA 7.0 software. To identify the CAEs in the *ZmDi19-5* promoter, the promoter sequence was analyzed using the PlantCARE (http://bioinformatics.psb.ugent.be/webtools/plantcare/html/, accessed on 2 November 2018) online tool [49], and the detected CAEs were further compared with the predicted results by the PlantRegMap database (http://plantregmap.gao-lab.org/binding_site_prediction.php, accessed on 20 June 2022) [50].

### 4.5. Transient Transformation in Tobacco

To determine the promoter activity of *ZmDi19-5* in regulating downstream genes, the 2000 bp promoter sequence was firstly subcloned into the pGreenII 0800-LUC vector with specific primers (pZmDi19-5-F and pZmDi19-5-R, Table S5) to construct the Pro_ZmDi19-5_:LUC fusion expression cassette by homologous recombination method. At the same time, the 35S promoter was amplified (35S-F and 35S-R, Table S5) and cloned into this vector to construct a 35S:LUC fusion expression cassette to function as a positive control. The constructed vectors were validated by sanger sequencing, and then transformed into the *Agrobacterium tumefaciens* GV3101 strain by freezing and thawing method. For the *Agrobacterium* transient transformation, the *Agrobacterium* harboring the constructed vector was cultured to OD_600_ = 1.5, and then centrifuged to collect the cultured bacteria. The transformation buffer containing 0.5 mol/L MES, 1 mol/L MgCl_2_, and 0.1 mol/L AS was used to re-suspend the bacteria. Six-week-old tobacco grown in the artificial climate room was used for the *Agrobacterium*-mediated transient transformation using the injection method.

### 4.6. Determination of LUC Activity and Identification of Promoter Core Region

The inoculated tobacco was placed in the dark for 36–48 h, and then LUC activity was analyzed using the NightShade LB 985 in vivo plant molecular imaging system (Berthold Technologies, Bad Wildbad, Germany). The LUC activity was determined using a Dual-Luciferase^®^ Reporter (DLR™) Assay System (Promega, USA) according to the manufacture’s protocol. The pGreenII 0800-LUC vector allows co-expression of LUC under the controlling of target promote and Renilla luciferase (REN) under the control of CaMV 35S promoter, which can be used for quantitative analysis of LUC activity through determining the ratio of LUC to REN. To identify the core region of promoter activity, the *ZmDi19-5* promoter was consecutively truncated from a 5′terminal into five regions according to the distribution of stress-related CAEs. The truncated fragment was amplified using corresponding primers (Table S5) and cloned into the pGreenII 0800-LUC vector by homologous recombination method, respectively. The LUC activity of different truncated promoter fragments was determined by *Agrobacterium*-mediated transient transformation with the same method.

### 4.7. Yeast One-Hybrid Library Screening

In order to screen the upstream regulatory proteins of *ZmDi19-5*, the identified core region of the 2000 bp promoter sequence was amplified (Pro_ZmDi19-5_-450-F and Pro_ZmDi19-5_-450-R, Table S5) and cloned into pAbAi vector (Clontech, CA, USA) by restriction enzyme digestion (HindIII and SacI) and the re-ligation method. The constructed pAbAi-Pro_ZmDi19-5_-450 was transformed into the yeast strain Y1HGold to function as the bait according to the manufacture’s protocol of the Matchmaker^TM^ Gold Yeast One-Hybrid Library Screening System (Clontech, CA, USA). Then, the positive yeast clones were cultivated on SD/-Ura medium with different concentrations of AbA at 30 °C to determine the final screening concentration. For Y1H preliminary screening, the library plasmid from leaves of maize inbred line B73 was transformed into yeast strain Y1HGold harboring pAbAi-Pro_ZmDi19-5_-450 construct, and then cultured on SD/-Leu plates containing 500 ng/mL AbA at 30 °C for 3-5 d. Then, nucleotide sequencing was performed for each positive clone using the isolated plasmid DNA. To explore the biological roles of the screened protein sequences, GO analysis was performed using ClusterProfiler [51], and functional prediction of these genes and their homologs were analyzed using MaizeGDB (https://www.maizegdb.org/, accessed on 1 June 2022), TRIR (https://www.arabidopsis.org/, accessed on 1 June 2022) and RiceData (https://www.ricedata.cn, accessed on 1 June 2022) online databases. Finally, the positive clones of the five proteins were selected and further tested on SD/-Leu plates containing 500 ng/mL AbA according to the functional annotations.

## 5. Conclusions

In conclusion, seven non-redundant Di19 family genes were identified in maize, and systematic analysis of these genes were performed by bioinformatics analysis. According to the phylogenetic relationships, the *ZmDi19-5* promoter was cloned and subsequently activity analysis was performed. The promoter activity of *ZmDi19-5* can be significantly induced by drought stress, suggesting the important roles of *ZmDi19-5* in response to drought stress. A 450 bp core region of *ZmDi19-5* promoter was identified and used for screening upstream regulatory proteins using Y1H system. A total of 28 proteins were obtained, and functional annotation indicated that some members were involved in photosynthesis, light response, and water transport related to biological processes. Importantly, five proteins showed strong binding activity to the promoter core region, which may function as the important upstream regulators of *ZmDi19-5* involved in the regulating of drought response.

## Data Availability

Not applicable.

## Supplementary Materials

The following supporting information can be downloaded at: https://www.mdpi.com/article/10.3390/ijms23137390/s1.
