# Supplementary material for: Identification and Characterization of the Core Region of ZmDi19-5 Promoter Activity and Its Upstream Regulatory Proteins
Source: Int J Mol Sci. 2022 Jul 2;23(13):7390. doi: 10.3390/ijms23137390 (PMC9267117; doi:10.3390/ijms23137390)
Supplement: Supplementary file 1 [file ijms-23-07390-s001.zip › Table S1.pdf]

Table S1. Detailed information of ZmDi19 genes in the maize genome

Gene Name	Transcript ID	ORF (bp)[a]	Introns[b]	Chr.[c]	Deduced polypeptide[d]		
					Length (aa)	MW (kDa)	pI
ZmDi19-1	Zm00001d011561_P003	744	4	8	247	27.12	5.14
ZmDi19-2	Zm00001d041965_P001	627	4	3	208	23.49	5.20
ZmDi19-3	Zm00001d043911_P001	744	4	3	247	27.33	4.96
ZmDi19-4	Zm00001d023808_P001	717	4	10	238	26.79	4.55
ZmDi19-5	Zm00001d016442_P007	720	4	5	239	27.24	6.67
ZmDi19-6	Zm00001d037966_P001	702	4	6	233	25.47	5.38
ZmDi19-7	Zm00001d038999_P001	690	4	6	229	25.31	5.38

[a]Length of open reading frame in base pairs.

[b]Number of introns of ZmDi19 genes.

[c]Chromosomal localization of ZmDi19 genes.

[d]Length (number of amino acids), molecular weight (kilodaltons), and isoelectric point (pI) of the deduced polypeptides.
