# Supplementary material for: Identification and Characterization of the Core Region of ZmDi19-5 Promoter Activity and Its Upstream Regulatory Proteins
Source: Int J Mol Sci. 2022 Jul 2;23(13):7390. doi: 10.3390/ijms23137390 (PMC9267117; doi:10.3390/ijms23137390)
Supplement: Supplementary file 1 [file ijms-23-07390-s001.zip › Table S2.pdf]

Table S2. Detailed information of the 15 motifs identified in ZmDi19 proteins.

Motif	Width	Conserved amino acid sequences
1	50	CPYCYEDHDVASLCAHLEEEHPYEPKAAPCPICSQRVTKDMINHITMQHG
2	35	IDSSLTSEEREQKRKQATGRATFVQGLLLSTLFGD
3	25	MDSEHWISRLAAAKRYYAAQLGHVD
4	40	RSSNAVATNISSDPLLSSFGLGFSSSDAPEPSKSASSIPD
5	21	YLFKNGHRSRRFIIPGSHAJS
6	15	RDLRGTHLQALLGGG
7	29	TEEVEMEIEDDGGLEMELEMALELGDATW
8	10	IRKETPVQPW
9	16	RSKFVRGLVLSLIFDD
10	6	KWRNKG
11	6	DVPGIG
12	19	YVRNPKDPAPDPFLSQFIC
13	6	NNWFKR
14	6	MDEEVR
15	7	YDLNMGI
