# Supplementary figures and images for: Identification and Characterization of the Core Region of ZmDi19-5 Promoter Activity and Its Upstream Regulatory Proteins
Source: Int J Mol Sci. 2022 Jul 2;23(13):7390. doi: 10.3390/ijms23137390 (PMC9267117; doi:10.3390/ijms23137390)

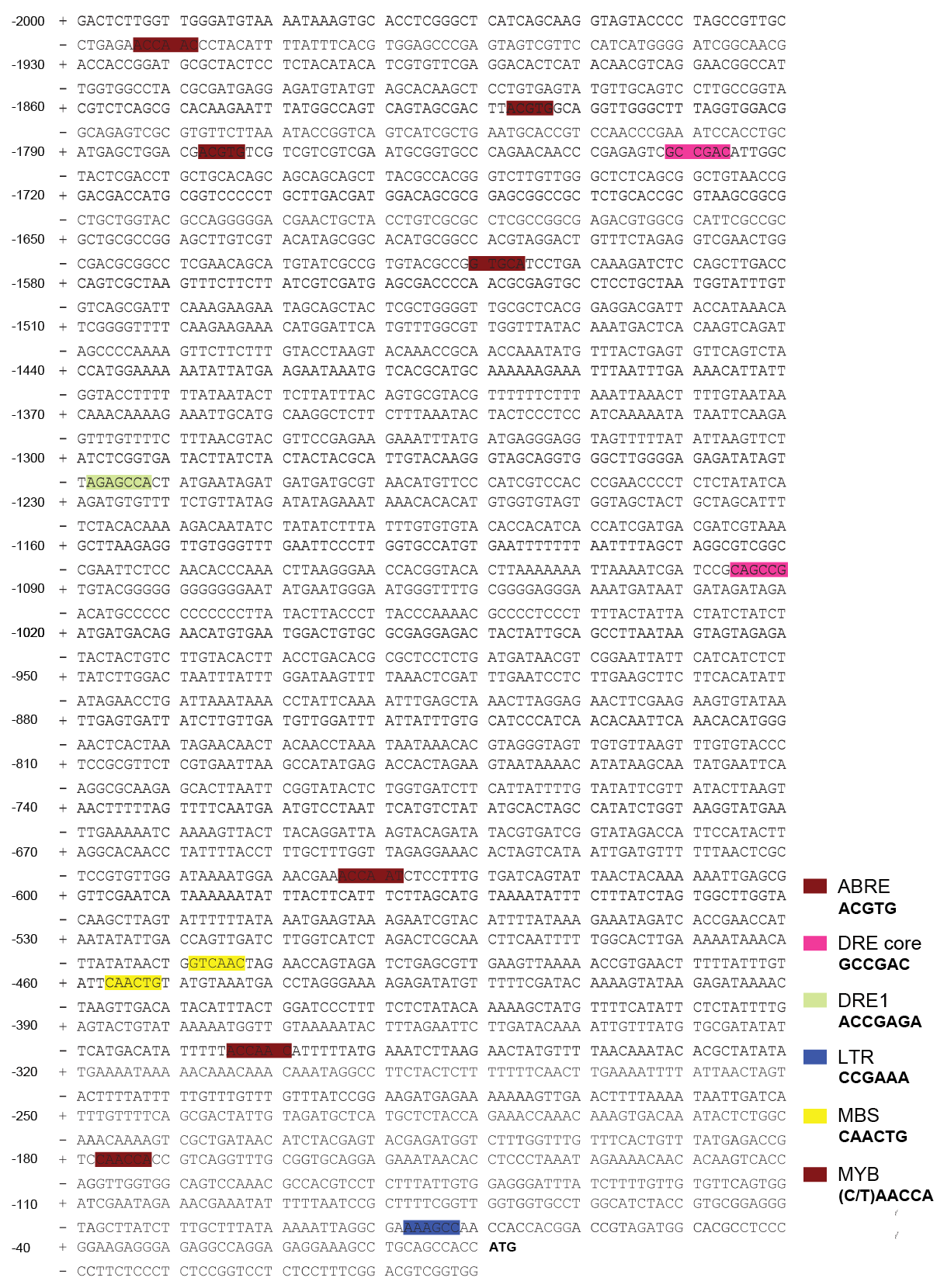

Supplement: Supplementary file 1 [file ijms-23-07390-s001.zip › Figure S1.tif]
